# Enhancing the Selectivity of Nitroso-R-Salt for the Determination of Co(II) in Lithium Bioleaching Recovery of Smartphone Batteries Using a Combinatorial Methodology Approach

**DOI:** 10.3390/nano15161264

**Published:** 2025-08-16

**Authors:** David Ricart, Antonio David Dorado, Mireia Baeza, Conxita Lao-Luque

**Affiliations:** 1 Department of Mining, Industrial and ICT Engineering, Escola Politècnica Superior d’Enginyeria de Manresa, Universitat Politècnica de Catalunya, Avinguda de les Bases de Manresa 61-73, 08240 Manresa, Spain; david.ricart.fort@upc.edu (D.R.); toni.dorado@upc.edu (A.D.D.); 2 GENOCOV Research Group, Department of Chemistry, Faculty of Science, Edifici C-Nord, Universitat Autònoma de Barcelona, Carrer dels Til·lers, 08193 Bellaterra, Spain

**Keywords:** Co(II), cobalt, nitroso-R-salt, combinatory analysis, batteries, bioleachate, leachate, UV-vis spectrophotometry

## Abstract

The selectivity of the colorimetric method for Co(II) determination using the nitroso-R-salt (NRS) in samples with complex matrices has been improved. Interferences caused by Cu(II), Fe(II), Fe(III), Mn(II), Al(III) and Ni(II) ions, which were present in the bioleach ate of lithium-ion batteries, have been solved through the sequential addition of masking agents: acetate, fluoride, ethylenediaminetetraacetic acid (EDTA), and strong acids (H_2_SO_4_). The absorbance of the NRS-Co(II) complex was typically measured at 525 nm, but it was also studied at 550 nm due to minimal interferences observed at 550 nm. The sequence of the masking agent’s addition showed a significant influence on the interference effect. The optimal sequence was sample, acetate–acetic acid buffer solution with dissolved fluoride, NRS, EDTA and H_2_SO_4_. The proposed method demonstrated robust performance at 550 nm, with a relative standard deviation (RSD) around 2%, and good accuracy (RV% around 100%). The limit of detection (LoD) was 0.1 mg L^−1^ and the limit of quantification (LoQ) was 0.3 mg L^−1^. The linear range extended up to 15 mg L^−1^ (R^2^ = 0.998). Real samples analyzed using the optimized method showed no significant differences when compared to results from atomic absorption spectroscopy, confirming its reliability.

## 1. Introduction

The increasing demand for lithium-ion batteries (LIBs), which are widely used in electronic devices, renewable energy and electrical vehicles, has driven the necessary recovery of valuable materials contained in batteries such as cobalt [1]. The demand for LIBs is expected to increase, with raw materials serving as the primary source of supply [2]. Due to their numerous advantages, including a low self-discharge rate, a long cycle life, a high energy density, and a lightweight design, LIBs are the optimal choice for a diverse range of applications, including electronic gadgets and electric mobility. Concurrently, the growing prevalence of LIBs has led to mounting pressure on geological deposits containing metals such as manganese, nickel, cobalt, lithium, and other metals. Consequently, in order to alleviate the pressure on geological reserves, it is of paramount importance to recover materials from discarded LIBs [3].

There are two major methods to recycle LIBs, pyrometallurgy and hydrometallurgy. Pyrometallurgy is a process whereby metals are extracted from other components by burning off electrolytes, binders, and polymers at high temperatures. Hydrometallurgy is a process that extracts metals from batteries by leaching them with acids and reducing agents [4]. Dai et al. [5] propose a novel method for recovering the metals in cathode materials from spent LIBs using a mixed iron salt composed of Fe_2_(SO_4_)_3_ (precipitant) and FeSO_4_ (reducing agent).

Recent hydrometallurgical methods for recovering metals from electronic waste and LIBs involve the activity of some microorganisms [6,7,8,9,10]. This process is called bioleaching and is being intensively researched as a promising and environmentally friendly method for recovering cobalt and other metals from obsolete LIBs [11]. In the bioleaching of LIBs, microorganisms or their metabolites are used to convert metal elements in the cathode into soluble ions in solution (leachate). The leachate contains a mixture of various metal ions, such as Li(I), Co(II), Ni(II), Mn(II) and Fe(III). To ensure the proper functioning of the bioleaching process, it is necessary to monitor the concentration variations of the metallic cations throughout the process. This can be performed automatically and in situ using a miniaturized flow injection analysis (FIA) [12,13].

There are several instrumental analytical methods to determine Co(II) in solution, such as atomic absorption spectrophotometry [14,15], fluorescence [16], thermal lens microscopy [17], potentiometry [18,19], chemiluminescence [20] and colorimetry [21,22,23,24,25,26]. FIA can use UV-Vis absorption as a detection method among other techniques [27,28]. The colorimetry strategies, which are easy to adapt to a FIA system [12,13], involve reaction of the metal ion to be determined with a chelating agent that forms a colored complex, the intensity of which is directly proportional to the metal concentration. The most used chelating agent for the cobalt colorimetric determination is the nitroso-R salt (NRS), which forms a red-orange complex [29]. The problem with this chelating agent is that it exhibits interferences with Fe(II), Fe(III), Cu(II), Ni(II) and many other metallic cations which are precisely found in bioleachates from batteries [24,30,31,32,33,34]. The low selectivity of NRS for Co(II) had led to the development of new chelating agents specific for Co(II) [35,36,37,38,39,40]. These new chelating agents resolve the selectivity problem but they are not commercially available; they need to be synthesized in the laboratory. This is quite expensive and requires stringent measures to ensure the purity of the final product. Thus, despite its limitations, this chelating agent is still the most widely used for Co(II) colorimetric determination.

Various studies have shown that method selectivity can be improved by adding masking agents that eliminate or significantly reduce interference from other metal ions. One noteworthy attempt to improve the selectivity of the colorimetric determination of Co(II) using NRS was reported by Shipman et al. [41]. The authors buffered the sample to pH 5.5 using a sodium acetate–acetic acid buffer, and NRS was added prior to heating the solution to boiling. Subsequently, concentrated nitric acid was introduced, and the solution was diluted after cooling. To counteract interferences, the authors explored different strategies, including the addition of potassium fluoride before NRS, preheating the buffered solution to enhance acetate complexation, alternated acidification and neutralization cycles after complex formation (referred to as “recycling”), and the use of an excess of NRS. According to the authors, fluoride and acetate masked Fe(III), Cu(II) and Fe(II), and fluoride ions precipitated the nickel removing their interference. Moreover, the excess of NRS removed the interference from Fe(II). However, subsequent studies have not been able to replicate or expand the results of this methodology, in their entirety.

Other authors investigated the ethylenediaminetetraacetic acid (EDTA) behavior. EDTA is an unspecific chelating agent that could compete with NRS and form stable complexes [42,43,44].

Nevertheless, there was a lack of agreement between studies. The outcomes of one study concluded that EDTA had no significant effect if it was used in low concentration and introduced after the complexes extraction using tetradecyldimethylbenzylammonium iodide on naphthalene in a packed column [42]. Results from another study were that EDTA avoids the formation of all complexes when adding it before NRS [43]. Other findings from another study showed that when EDTA was added before, no interferences were observed from Fe(III) and Ni(II), and EDTA was used to remove the excess of NRS in an anion-exchanger [45]. However, a better description was provided by Adam J. & Přibil R. [44]; when EDTA is added before NRS, it prevents the cobalt reaction, whereas if added after NRS, the Co(II)-NRS complex forms and cannot be disrupted by EDTA even at boiling temperature. This behavior cannot be explained by the formation constant with EDTA (logβ_Co-EDTA_ = 16.3 [46]) being higher than that of NRS (logβ_Co-NRS_ = 13.3 [47]) as it was proposed by the authors of this study [43].

The effect of pH was also studied showing that in this case the results were more consistent than the EDTA results. The velocity of reaction exhibited significant dependence on pH. At low pHs the reaction was slower than at high pHs [48,49], even the non-formation of complexes to pH below 3 [48,50]. The optimal pH for the reaction was 5.5 [51]. Nonetheless, when the reaction between Co(II) and NRS was completed, acid and heating can be used to remove interferents from many cations such as Ni(II), Fe(III), Cu(II), and Zn(II) [34,41,44]. Yet, the main disadvantage is that after the treatment with acid a large amount of free reagent remains in the solution and has a considerable absorbance [44].

In the same sense, some authors established that when acetate and fluoride were used as masking agents, they should be added before NRS [41,42,44]. Still, there were inconsistencies between results, since the findings of Shipmen et al. [41] showed that fluoride can mask Fe(III) and Cu(II), which were not observed by Puri, B.K. & Balani, S. [42].

In the majority of these studies, preconcentration or extraction was a required step [42,43,44,45].

This implies that the sequence of chelating compound addition affected the result. NRS was unable to dissociate the Co(II)-EDTA complex and vice versa [44]. Moreover, when acetate and fluoride were used as masking agents, they should be added before NRS [41,44] and acid should be added after reaction [34,41,44]. It is important to notice that there have been approaches to studying the order addition before [34,44], but they did not make a thorough study about it.

All these studies avoid the interference of some metallic cations, but there is no described method dealing with the elimination of all the interferences that can be found in a battery bioleach (Cu(II), Fe(II), Fe(III), Mn(II), Al(III) and Ni(III)), nor with the order of addition of all these masking agents when there are used together.

In this work, for the first time, all those masking agents (EDTA, fluoride, acetate, acetic acid and strong acid) have been used together in order to avoid interferences caused by cations (Cu(II), Mn(II), Fe(II), Fe(III), Al(III) and Ni(II)), usually present in a bioleachate from LIBs, enhancing the selectivity of the method without the necessity of boiling or a previous preconcentrating step. Furthermore, a detailed study on the influence of the order of addition of each one on the colorimetric determination of cobalt with NRS has been carried out. Additionally, hydroxylamine was investigated as a means of avoiding interferences, reducing the oxidation state of cationic metals, and decreasing the variety of cations, such as the reduction of Fe(III) to Fe(II). Also, we tested if Cu(I) interferes as Cu(II), since no study has been found to study the effect of Cu(I).

## 2. Chemicals and Methods

### 2.1. Chemicals, Reagents and Equipment

The Co(II) was used as CoSO_4_·7H_2_O (99%) from Panreac (Castellar del Vallès, Spain), Al(III) as Al_2_(SO_4_)_3_·18H_2_O (Al_2_(SO_4_)_3_ 51–59%) from Scharlau (Barcelona, Spain), Cu(II) as CuSO_4_·5H_2_O (99%) from Labkem (Barcelona, Spain), Fe(II) as FeSO_4_·7H_2_O (99%) from Chem-lab (Zedelgem, Belgium), Fe(III) as FeNH_4_(SO_4_)_2_·12H_2_O (99%) from Scharlau (Polinya, Spain), Ni(II) as NiSO_4_·6H_2_O (≥99%) and Mn(II) as MnSO_4_·H_2_O (≥99%) from Panreac (Castellar del Vallés, Barcelona).

1,10-phenanthroline monohydrate from Labkem (Barcelona, Spain) was used to prepare a 0.1% *w/v* (0.005 mol L^−1^) solution.

Hydroxylamine hydrochloride (98%) from Panreac (Castellar del Vallès, Spain) was used to prepare a 10% *w/v* (1.4 mol L^−1^) aqueous solution.

The EDTA solution of 0.05 mol L^−1^ and 0.1 mol L^−1^ were obtained from Scharlab (Sentmenat, Spain).

A 33.3 g L^−1^ (0.57 mol L^−1^) solution of potassium fluoride (KF) (≥99%) from Fisher Scientific (Geel, Belgium) was prepared to study its masking effects. This concentration was selected to ensure that, upon adding 4 mL to a final volume of 10 mL in the sample preparation, the resulting KF concentration would be 13.2 g L^−1^ (0.23 mol L^−1^), matching the conditions described in the study of Shipmen et al. [41].

A 0.022 mol L^−1^ solution of acetate was prepared dissolving 3 g L^−1^ of sodium acetate trihydrate (99+%) from T3Q (Sentmenat, Spain). Also, acetate–acetic acid buffer was prepared dissolving 0.022 mol L^−1^ of sodium acetate trihydrate and 0.007 mol L^−1^ of acetic acid (99.8%) from Panreac (Castellar del Vallès, Barcelona).

H_2_SO_4_ (95–98%) was from Panreac (Castellar del Vallès, Spain) and utilized to prepare an aqueous solution with at pH = 1.8. This solution was used to prepare the metal interference solutions and to dilute the samples. In addition, H_2_SO_4_ solutions ranging from 5% *v/v* to 25% *v/v* were prepared for the interference study. Also, HCl 37% from Panreac (Castellar del Vallès, Spain) was used.

Three stock solutions of NRS disodium from Fluka AG (Buchs, Switzerland) were prepared: one at a concentration of 1.68 × 10^−4^ mol L^−1^, following the same concentration used by Issa et al. [24]; another at a concentration of 4.0 g L^−1^ (0.0106 mol L^−1^); and a third containing 4.0 g L^−1^ (0.0106 mol L^−1^) of NRS and 3.0 g L^−1^ (0.022 mol L^−1^) of sodium acetate trihydrate. NRS was stored in opaque containers and dark environments.

All solutions were prepared with deionized water from Milli-Q system (Millipore, Billerica, MA, USA).

Absorbance was measured at 525 nm in the initial experiments, despite the NRS-Co(II) complex having a maximum absorption at 415 nm. This is because the NRS reagent does not absorb at 525 nm [41]. However, from Section 3.5 onward, measurements were conducted at 550 nm and 710 nm to assess Fe(II) interference based on Section 3.4 findings. All measurements were carried out using a UV-Vis spectrophotometer (PerkinElmer, PerkinElmer Lambda 25, Shelton, CT, USA).

### 2.2. Interference Study

Interferences in the colorimetric determination of Co(II) caused by the presence of various metal ions were addressed by adding masking agents (H_2_SO_4_ or HCl, KF, EDTA, acetate, and hydroxylamine). A series of experiments were conducted to study the effect of reagent addition order, aiming to determine the optimal sequence for the selective detection of Co(II). However, as the procedure involves six reagents in total, including NRS, testing all 720 (6!) possible combinations for each metal was not feasible. Additionally, the influence of reagent concentrations on the method’s selectivity was also examined, further increasing the complexity of the study. For this reason, a stepwise approach was adopted, meaning that experimental conditions were adjusted based on the results of previous experiments, focusing solely on the most promising configurations. Consequently, the experimental procedures and results are presented in the order of execution.

#### 2.2.1. Experimentation of Each Reagent Separately

The individual effects of various masking agents—acetate, EDTA, strong acids, hydroxylamine, and fluoride—were evaluated for their ability to minimize interferences caused by Fe(II), Fe(III), and Cu(II), as these metals generate the most significant and challenging interferences [41].

For this purpose, solutions containing 800 mg L^−1^ (0.013 mol L^−1^) of Cu(II), 320 mg L^−1^ (0.0057 mol L^−1^) of Fe(III), and 320 mg L^−1^ (0.0057 mol L^−1^) of Fe(II) were prepared. Aliquots of 0.5 mL from each metal solution were mixed with 0.1 mL of a 160 mg L^−1^ (0.0027 mol L^−1^) Co(II) solution. Subsequently, 2 mL of the NRS (reagent) were added. The masking agents were then introduced as follows: 4 mL of fluoride (based on Shipman’s method [41]), 2 mL of EDTA, and 7 mL of acetate, ensuring an excess of EDTA and acetate in the mixtures. Additionally, 1 mL of hydroxylamine and 4 mL of strong acids (37% *v/v* HCl and 25% *v/v* H_2_SO_4_) were used. Finally, all test solutions were diluted to a final volume of 10 mL with Milli-Q water. Therefore, the concentration of cations and reagents in the mixture were: 40 mg L^−1^ (6.3 × 10^−4^ mol L^−1^) of Cu(II), 16 mg L^−1^ (2.9 × 10^−4^ mol L^−1^) of Fe(II) and Fe(III), 1.6 mg L^−1^ (2.7 × 10^−5^ mol L^−1^) of Co(II), 0.23 mol L^−1^ of KF, 0.01 mol L^−1^ of EDTA, 0.031 mol L^−1^ of acetate and 0.14 mol L^−1^ of hydroxylamine. For NRS, two concentrations were 0.0021 mol L^−1^ and 3.36 × 10^−5^ mol L^−1^ in the mixture.

The effect of each masking agent was examined by comparing the outcomes when added before and after NRS, as well as in its absence.

#### 2.2.2. Combinatorial Experimentation to Inferences Studies

In this section, the ability of the combined use of masking agents—fluoride, EDTA, acetate and hydroxylamine—to reduce Cu(II), Fe(II) and Fe(III) interferences in the colorimetric determination of Co(II) using NRS has been evaluated.

The experiments were designed based on the results obtained in the previous Section 3.1. So, from this point onward, the NRS stock solution was prepared 4 g L^−1^ (0.0106 mol L^−1^) (justified in Section 3.1.1) and 3 g L^−1^ (0.022 mol L^−1^) of sodium acetate trihydrate (justified in Section 3.1.4). Additionally, 33.3 g L^−1^ KF (0.57 mol L^−1^) was dissolved in an acetate–acetic acid buffer solution (0.022 mol L^−1^ of acetate and 0.007 mol L^−1^ of acetic acid), which maintained pH near optimal, justified in Section 3.1.7. Also, strong acid was not used for combinatorial experiments (justified in Section 3.1.2).

As a result, four distinct reagent solutions were defined, reducing the complexity of the subsequent combinatorial experiments from 720 (6!) possible combinations to 24 (4!). These experiments aimed to assess the influence of the order of addition of reagents on the interferences caused by Cu(II) and Fe(III).

To further minimize interferences in the colorimetric determination of Co(II), absorption spectra were recorded between 470 and 800 nm for the NRS complexes of Fe(II), Fe(III), Al(III), Ni(II), Cu(II), and Mn(II). The aim was to identify wavelengths at which these interfering metal complexes exhibited minimal absorbance, while the Co(II)–NRS complex maintained a strong and distinct signal.

##### Study of Cu(II) Interferences

1. Combinatorial experimental:

Cu(II) was selected as a starting point because according to Shipmen et al. [41] it is one of the main sources of interference in the determination of Co(II). Additionally, Cu(II) is very abundant in lithium-ion batteries utilized in electric mobility such as cars or scooters [52,53,54] and therefore, in the bioleachate of the present work. 40 mg L^−1^ of Cu(II) (6.3 × 10^−4^ mol L^−1^) and 1.6 mg L^−1^ (2.7 × 10^−5^ mol L^−1^) of Co(II) were used to maintain the same concentration rate described in the work of Shipmen et al. [41].

Using Minitab 19 software (Minitab Inc., State College, PA, USA) 24 possible combinations sequences were obtained and shown in Table S1. However, taking into account the inability of NRS to displace the Co(II) from the EDTA-metal complex explained by Adam and Přibil [44], combinations which EDTA precedes NRS were not tested. The combinations evaluated are shown in Table 1.

In total, 0.5 mL of 800 mg L^−1^ (0.013 mol L^−1^) Cu(II) solution and 0.1 mL of 160 mg L^−1^ (0.0027 mol L^−1^) of Co(II) were mixed, then reagents were added. The volumes of each reagent solution were: 1 mL of hydroxylamine (0.14 mol L^−1^ in the mixture), 2 mL of NRS (0.0021 mol L^−1^ in the mixture), 4 mL of KF in acetate–acetic acid buffer (0.23 mol L^−1^ of KF, 0.0088 mol L^−1^ of acetate, 0.0028 mol L^−1^ of acetic acid in the mixture), and 2 mL of 0.05 mol L^−1^ EDTA (0.01 mol L^−1^ in the mixture). Finally, Milli-Q water was added to reach a final volume of 10 mL. In all cases, the sample was always added first. These volumes were chosen to ensure EDTA and NRS were in excess, hydroxylamine was chosen according to the method used in the laboratory [55] and KF in acetate–acetic acid buffer was explained in Section 2.1 and Section 3.1.7.

Each combination was tested with and without Cu(II), and each test was replicated three times. The blank of each sequence tested consisted of adding all the reagents in the same order but without any cationic metal, the sample was 1 mL of Milli-Q water. Finally, the absorbance value obtained with and without Cu(II) for a same sequence was subtracted, and the relative error was calculated. The smaller the relative error, the less Cu(II) interfered with the measurement.

2. Optimization of reagent concentrations using Taguchi experimental design:

The influence of reagent concentrations on the suppression of interferences was evaluated using a Taguchi experimental design, implemented with Minitab 19 software. For this purpose, one of the most promising reagent addition sequences identified for minimizing Cu(II) interference—Sequence 8, as described in Section 3.2.1—was selected as the basis for the study.

Four concentration levels (0, low, medium, and high) were tested for all reagents except for NRS, which was evaluated at two levels (low and medium). The reagent concentrations corresponding to each level are detailed in Table S2. The combinations of concentration levels used in experimental assays are detailed in Table S3.

##### Study of Fe(III) Interferences

Another metallic ion abundant in the bioleachate obtained from batteries is the Fe(III). This ion is obtained by oxidation of Fe(II) carried out by the microorganisms.

Based on results obtained in the Cu(II) interference study (Section 3.2.1), twelve different reagent addition sequences were explored for Fe(III), using the same volumes, Co(II) concentration and reagent concentration of reagents as in the copper combinatorial study. The tested sequences are summarized in Table 2. For each trial, absorbances values were recorded both with and without Fe(III). The difference between these values was used to calculate the relative error, with lower relative errors indicating reduced interference from Fe(III) in the Co(II) determination.

The final concentration of Fe(III) in the prepared mixture was 16 mg L^−1^ (2.9 × 10^−4^ mol L^−1^), achieved by adding 0.5 mL of a 320 mg L^−1^ (0.0057 mol L^−1^) Fe(III) stock solution.

##### Study of Fe(II) Interferences

The interference caused by Fe(II) is due to its formation of a complex with NRS, which has a significant absorption across the entire visible zone spectrum, particularly at 710 and 520 nm, where absorption processes take place and several peaks are observed [48].

Notably, the Fe(II)-NRS complex produces the most persistent interference signal, which proves particularly challenging to eliminate [41].

Experiments were conducted in the same manner as described in Chemicals and Methods, Section Study of Fe(III) Interferences, but the volumes of the reagents added were adjusted based on the results obtained in the previous experimental sections. Volumes were optimized based on two key considerations: (1) results from previous experimental sections, and (2) the need to test varying volumes of KF in acetate–acetic acid buffer, since EDTA cannot effectively suppress the Fe(II) signal (explained in Section 3.1.6). Specific modifications included, NRS volume: fixed at 1 mL (0.00106 mol L^−1^ in the mixture), hydroxylamine was removed, EDTA concentration: adjusted from 2 mL of 0.05 mol L^−1^ to 1 mL of 0.1 mol L^−1^ (0.01 mol L^−1^ in the mixture). These volume reductions for EDTA and NRS allowed for increased KF in acetate–acetic acid buffer, as KF concentration represented the final adjustable parameter for mitigating Fe(II) interference. To systematically evaluate this effect, KF volumes of 4, 5, 6, and 7 mL were tested against Fe(II) solutions. Therefore, the concentrations of KF in the preparation were 0.23 mol L^−1^, 0.29 mol L^−1^, 0.34 mol L^−1^ and 0.40 mol L^−1^, respectively. For acetate were 0.0088 mol L^−1^, 0.011 mol L^−1^, 0.013 mol L^−1^ and 0.015 mol L^−1^, respectively. For acetic acid were 0.0028 mol L^−1^, 0.0035 mol L^−1^, 0.0042 mol L^−1^ and 0.0049 mol L^−1^, respectively. Cobalt was deliberately omitted from these tests to isolate the absorbance contribution from Fe(II). Moreover, Fe concentration was 20 mg L^−1^ (3.6 × 10^−4^ mol L^−1^) Fe(II) matching actual bioleachate concentration.

##### Interferences Study with Combined Metals Ions Present in Batteries Bioleachates

Employing the optimal reagent addition sequence and concentrations established in the individual interference tests, a comprehensive interference assessment was conducted with all relevant metal ions separately. For each metal, three concentration levels—low, medium, and high—were evaluated. The specific concentrations used are detailed in Table 3. The medium level was equal to the value in a battery bioleachate in our lab from mobile phones [52,53,54]. The low concentration was set 10 mg L^−1^ lower (except for Mn, which was 5 mg L^−1^ lower), while the high concentration was 10 mg L^−1^ higher. The concentrations of Fe(II) and Fe(III) were selected based on the concentration of the medium (9000 mg L^−1^) feeding the microorganisms in the bioreactor, which when the sample is prepared for measurement is diluted 450 times, thus becoming 20 mg L^−1^ total iron. Each test was performed in triplicate.

### 2.3. Validation of Method

The analytical method was validated through the determination of repeatability, linearity, accuracy, detection and quantification limits. The study of repeatability was conducted by analyzing two different samples, which contain only Co(II) (2.5 and 10 mg L^−1^), six times each, then the variation coefficient was calculated and compared with those proposed by Horwith [56]. The determination of the linear range was calculated by measuring calibration solutions ranging from 1 to 20 mg L^−1^. Regarding limit of detection (LoD) and limit of quantification (LoQ), they were determined by measuring the signal of five blanks and calculated through signal-to-noise approach using the formula (X + 3S_b_)/S and (X + 10S_b_)/S, respectively, where X represents the mean of the blank measured, S_b_ is the standard deviation of the blank mean, and S is the slope of the calibration curve [57].

Furthermore, to complete the validation study, Co(II) was determined in real samples, and the results were compared with those obtained by atomic absorption spectroscopy (PerkinElmer, PinAAcle 500, Shelton, CT, USA). The analyzed real samples were bioreactor medium, water from the Agulla reservoir in Manresa (Manresa, Spain), bioleachates from smartphone PCBs and bioleachates from batteries of bikes and scooters.

The bioreactor medium, bioleachates from smartphone PCBs and water from the Agulla reservoir samples were spiked with 3, 4 and 7 mg L^−1^; 2, 3.5 and 6 mg L^−1^ of Co(II); 1.5, 8 and 9 mg L^−1^, respectively, as they did not contain Co(II) themselves.

Accuracy was determined by adding a 5.5 mg L^−1^ Co(II) standard to the sample and calculating the recovery percentage (RV%) in bioleachate from bicycle and scooter batteries. To evaluate three different concentrations, the leachate was diluted 270, 360 and 450 times, resulting in three different samples.

## 3. Results and Discussion

### 3.1. Examination of Individual Reagents

A series of experiments were conducted to analyze and evaluate the individual contribution of the six reagents which were NRS, KF, acetate, hydroxylamine, strong acid (H_2_SO_4_ or HCl), and EDTA in the development of the Co(II) determination method. The primary objective of these tests was to analyze the effect of each reagent to better understand its specific influence on the method. The results are presented below and summarized in Table 4.

#### 3.1.1. Nitroso-R-Salt

The initial experiments focused on examining the coordination of NRS with Co(II) as well as its interaction with other interfering metals ions. NRS was tested in the presence of various metal cations. Furthermore, the potential of using excess NRS to minimize Fe(II) interference, as commented in [41], was investigated.

NRS formed distinctly colored complexes with several metals cations commonly present in the battery bioleachate: Ni(II)-NRS yielded an orange complex, Fe(II)-NRS was green, Co(II)-NRS was red-orange and Cu(II)-NRS was brown as illustrated in Figure S1. Meanwhile, Fe(III) exhibited a unique pH-dependent behavior in its interaction with NRS. Fe(III)-NRS was brown in acidic medium and presented the same Fe(II)-NRS green in neutral medium. In Section 3.1.2 this behavior is explained. Similar colors were observed by Adam J. & Přibil R. [44].

Regarding NRS concentration, an initial test using 1.68 × 10^−4^ mol L^−1^ previously reported by Issa et al. [24] was found to be insufficient, since Co(II) concentrations in the bioleachate could reach up to 5.72 × 10^−4^ mol L^−1^. Considering the 1:3 metal-to-ligand molar ratio required for complete complex formation [58], a higher NRS concentration (4 g L^−1^, 0.0106 mol L^−1^ in the stock solution) was necessary to ensure it. Furthermore, increasing the NRS concentration did not mitigate Fe(II) interference, in contrast to the observations reported by Shipman et al. [41]. This highlights the necessity of incorporating specific masking agents—rather than relying solely on stoichiometric excess—to suppress interference from Fe(II).

An additional observation was the gradual degradation of NRS over time. Degradation was observed through the appearance of suspended particles between 24 and 48 h. Within a week, the particles become a soft, agglomerated precipitate at the bottom of the storage container. This degradation compromised the reliability and reproducibility of the analyses. Moreover, NRS degraded more quickly and formed larger aggregates in acidic environments. In order to avoid it, 3 g L^−1^ (0.0022 mol L^−1^) of sodium acetate trihydrate was dissolved in the NRS solution to increase the pH to 6. A deeper explanation about the use of acetate is in Section 3.1.4. Additionally, the stability of NRS significantly improved when the solution was stored in opaque containers or stored under dark conditions. The room temperature was around 25 °C.

#### 3.1.2. Strong Acids and pH-Dependency

Tests conducted with HCl and H_2_SO_4_ revealed no significant differences in the results, indicating that the determining factor was the pH value, rather than the specific acid used. As such, the discussion focuses on the role of pH in the complexation and stability of the Co(II)–NRS complex. As mentioned earlier, the complexation reaction progresses very slowly under strongly acidic conditions [48,49]. Figure S2 shows the time reaction of formation Co(II)-NRS at pH = 2. Likewise, other cations, except for Fe(III), exhibited analogous complexation behavior with NRS under these conditions. These results were inconsistent with results from other studies which said the non-formation of Fe(II)-NRS at pH values lower than 3 [48,50]. On the contrary, at near-optimal pH (≈5.5) the coordination reaction occurs rapidly and efficiently, in agreement with previous studies [34,41,42,43,44,45,48,49,50,51].

It is important to highlight that Fe(III) exhibited a unique pH-dependent behavior in its interaction with NRS. In acidic media, the Fe(III)–NRS complex formed rapidly, producing a brown coloration. Similar results were obtained before [44,59]. In contrast, under neutral conditions, the complex showed a green hue similar to that of the Fe(II)–NRS complex. To confirm that this shift was not caused by redox reactions—as previously speculated by Shipman et al. [41]—control experiments were conducted. A solution containing only Fe(III) was treated with 1,10-phenanthroline and an excess of acetate. No formation of the Fe(II)–phenanthroline complex was observed, confirming that no reduction of Fe(III) to Fe(II) had occurred. Therefore, the spectral absorbance of complex Fe(III)-NRS had a dependence with pH.

However, if the pH is drastically reduced after the complexes are formed (using 37% HCl or 25% H_2_SO_4_; adding 4 mL or more) complexes decomposed whereas the Co(II)-NRS complex remains stable, achieving high selectivity in Co(II) determination. The same was observed in previous studies [34,41,44,49]. The decomposition occurred instantaneously upon acid addition, except for Fe(II) which required significantly more time, as explained in Section 3.6. Indeed, it has been demonstrated that below a certain acidic pH threshold, not only the interfering complexes did not form but also even dissociated if already formed. Unfortunately, this critical pH value remains undetermined at this stage.

Nevertheless, it is important to point out that although the use of concentrated acids significantly improves Co(II) selectivity by selectively destabilizing competing metal complexes, this approach presents practical limitations. One was expressed by Adam J. & Přibil R. [44] and explained in the introduction. Another reason identified in this study was the fact that, in some cases, the required acid volume and concentration exceeded safe or feasible limits for the reaction mixture. To illustrate this point, the following example is provided: the addition of 2 mL of sample, 2 mL of NRS, and 6 mL of HCl at 37% *v/v* results in a residual signal from 600 mg L^−1^ (0.0094 mol L^−1^) of Cu(II). Additionally, the neutralization of such highly acidic mixtures could lead to vigorous exothermic reactions, resulting in rapid temperature increase and spattering, which were a security risk. The use of alternative masking agents could have prevented these occurrences.

Due to these drawbacks, this strategy was excluded from the combinatorial optimization experiments, reducing the number of reagent sequence permutations from 720 (6!) to 120 (5!).

#### 3.1.3. Hydroxylamine

In the case of hydroxylamine, no improvement in the method’s selectivity was observed. On the contrary, the reduction of Fe(III) to Fe(II) and Cu(II) to Cu(I) not only failed to eliminate interference but also exacerbated it, since Fe(II) caused stronger interference than Fe(III). Furthermore, the Cu(I)-NRS and Cu(II)-NRS complexes exhibited identical signals.

#### 3.1.4. Acetate

Acetate was initially evaluated for its potential as a masking agent, and its effectiveness was assessed based on the sequence of addition—either before or after NRS. Additionally, its role as a pH regulator was investigated, given that pH is a key factor influencing the efficiency of the coordination reaction between Co(II) and NRS.

The results revealed that acetate was unable to mask Cu(II) or Fe(II), in agreement with [42] and inconsistent with [41]. However, acetate can mask Fe(III), provided it was added prior to NRS. Under these conditions, the formation of the Fe(III)–NRS complex was slow, yielding a green color of reduced intensity that stabilized after a few hours.

Although acetate was an ineffective masking agent, with only temporary masking capabilities for Fe(III), it demonstrated utility in other roles. Specifically, the coordination reaction with metals was typically slow; however, when acetate was added after NRS, the reaction accelerates and completes promptly. Similarly, adding acetate before NRS ensures rapid completion of the reaction. To optimize the reaction efficiency, a stock solution containing 4 g L^−1^ (0.0106 mol L^−1^) NRS and 3 g L^−1^ (0.022 mol L^−1^) sodium acetate trihydrate was used hereafter. Trials with 1 g L^−1^ (0.0073 mol L^−1^) and 3 g L^−1^ sodium acetate trihydrate confirmed that 1 g L^−1^ was insufficient as the velocity reaction is not sufficiently rapid. This can lead to issues with reproducibility if the EDTA terminates the reaction too soon which is explained in Section 3.1.6. Furthermore, it was necessary to use more acetate to decelerate and minimize the degradation of NRS.

Additionally, acetate, when combined with acetic acid, acts as a buffer solution. As previously noted, maintaining an appropriate pH is critical for the reaction. Consequently, although acetate was a poor masking agent, temporarily masking Fe(III), it plays a crucial role in pH regulation. This ensures optimal conditions for the coordination reaction between NRS and Co(II). Acetate will continue to be utilized both in the preparation of acetate–acetic acid buffer solutions and as a component in the NRS stock solution to support reaction efficiency and long-term stability.

#### 3.1.5. Fluoride

To investigate the role of fluoride in ensuring the selectivity of Co(II) determination with NRS, experiments were conducted to examine the effect of the addition order by introducing fluoride either before or after the NRS. This approach aimed to evaluate fluoride’s contribution to enhancing the selectivity of the determination.

The results showed that fluoride was highly effective at masking both Fe(II) and Fe(III) when added prior to NRS. Similar results were obtained in earlier studies [41,42,44,48,50]. This masking effect was observed even at concentrations as high as 16 mg L^−1^ (2.9 × 10^−4^ mol L^−1^) of Fe(III) and from up to 7.5 mg L^−1^ (1.3 × 10^−4^ mol L^−1^) of Fe(II) was found to persist for over 12 h. Conversely, when fluoride was added after NRS, it was unable to break the complexes already formed between NRS and the metals. Regarding Cu(II) and Ni(II), their interaction with NRS was not significantly affected by the presence of fluoride, regardless of the order of addition, contrary to findings of this study [41].

These findings confirm that fluoride is a selective and durable masking agent for iron species, provided it is introduced before NRS, but it does not influence the coordination behavior of Cu(II) and Ni(II). Furthermore, fluoride showed no measurable effect on both pH and Co(II)-NRS formation. Tests with 15 mg L^−1^ (2.5 × 10^−4^ mol L^−1^) Co(II) solutions yielded identical absorbance values: 3.27 ± 0.05 (with fluoride) versus 3.28 ± 0.02 (without fluoride).

#### 3.1.6. EDTA

A series of experiments were carried out to evaluate the role of ethylenediaminetetraacetic acid (EDTA) in the selective colorimetric determination of Co(II) using NRS. As with fluoride, the effect of the order of reagent addition was examined, testing whether EDTA was added before or after NRS. The objective was to assess its ability to mitigate metal interferences and enhance method selectivity.

The results are consistent with those of previous studies [34,44]. EDTA, a non-specific chelating agent, forms coordination complexes with all the metals present. Once a metal is chelated by EDTA, NRS cannot react with it, as it cannot break the coordination bonds formed by EDTA. However, when NRS is introduced first and EDTA is added subsequently, EDTA disrupts the coordination bonds formed by NRS, except for those with Co(II) and, to a lesser extent, those with Fe(II). At a concentration of 0.016 mol L^−1^, EDTA has been shown to mitigate interference from up to 16 mg L^−1^ (2.9 × 10^−4^ mol L^−1^) of Fe(III) and more than to 40 mg L^−1^ (6.3 × 10^−4^ mol L^−1^) of Cu(II). In the case of Fe(II), as shown in Figure S3, EDTA reduces the signal but is unable to completely eliminate it, even at concentrations as low as 1 mg L^−1^ (1.8 × 10^−5^ mol L^−1^).

However, it is important to add EDTA after the complexation reaction was finished as upon the addition of EDTA, all coordination reactions involving NRS were immediately terminated. EDTA prematurely halted the reaction, potentially leading to erroneous conclusions. These results underscore the strategic importance of reagent sequencing.

#### 3.1.7. Combined Use of Acetate and Fluoride

The combined use of acetate–acetic acid buffer and fluoride was evaluated to optimize reagent addition in the Co(II)–NRS colorimetric method. Specifically, the sequence of addition was tested by introducing the buffer either before or after the fluoride, while ensuring that both were always added prior to NRS. The results showed no significant differences, leading to the decision to dissolve KF directly in the acetate–acetic acid buffer. This approach simplifies pH control and ensures optimal conditions during the reaction, avoiding potential pH fluctuations that could occur if KF were dissolved in Milli-Q water.

Since acetate lacks significant masking properties, its primary role was to control pH. Combining both reagents into a single solution allows the required concentrations for the final sample preparation to be maintained without increasing the concentrations in their respective stock solutions. This was particularly important given the total volume for sample preparation was 10 mL, with predefined proportions such as 2 mL of NRS, among others.

Furthermore, integrating the buffer and KF into a single solution significantly reduces experimental complexity. By reducing the number of solutions from five to four, the possible combinations decrease from 120 (5!) to 24 (4!), simplifying experimental design and execution without compromising the efficacy or accuracy of the combinatorial experiment.

### 3.2. Study of Cu(II) Interferences

#### 3.2.1. Combinatory Results

The effect of reagent addition order on the selectivity of Co(II) determination in the presence of Cu(II) was evaluated using 15 different reagent sequences. Each sequence involved the combination of hydroxylamine, NRS, EDTA, and a KF in acetate–acetic acid buffer, with all reagents added prior to measurement. The concentrations used were 1.6 mg·L^−1^ (2.7 × 10^−5^ mol L^−1^) Co(II) and 40 mg·L^−1^ (6.3 × 10^−4^ mol L^−1^) Cu(II), and all tests were conducted in triplicate with and without Cu(II) to assess interference. Table 5 summarizes the absorbance values at 525 nm, along with the absolute and relative errors.

The results demonstrate that the sequence of reagent addition significantly influences the extent of Cu(II) interference. Relative errors ranged from as low as 1.4% to as high as 21.4%, indicating substantial variability depending on the order.

The lowest relative errors—indicating minimal interference—were obtained in sequences 8 (N–H–E–B) and 10 (N–B–E–H), both showing a relative error of just 1.4%, followed by sequence 11 (N–E–H–B) with 3.3%, and sequence 2 (H–N–E–B) with 5.3%. These results suggest that adding NRS early, followed by EDTA and then the KF in acetate–acetic acid buffer, helps preserve the Co(II)–NRS complex while minimizing Cu(II) interference. On the other hand, sequences 1 (H–N–B–E), 12 (N–E–B–H), and 13 (B–H–N–E) exhibited the highest relative errors, at 21.4%, 19.0%, and 21.0%, respectively. As it can be seen, in general, adding NRS first or second, followed by EDTA and/or KF in acetate–acetic acid buffer, leads to lower interference. In contrast, sequences where the KF in acetate–acetic acid buffer was added too early (before NRS) tend to result in higher relative errors. Additionally, it was observed during the experiment that the addition of hydroxylamine was counterproductive.

These findings underscore the importance of reagent sequencing in maximizing selectivity. The use of EDTA and fluoride as masking agents is clearly effective—but only when timed correctly relative to NRS addition. The optimal sequences identified here will guide the final method design. The sequences 8 and 10 presented the lowest relative error. For the study of effect of reactant concentration made in Section 3.2.2, sequence 8 was selected since its standard deviation was lower than sequence 10.

#### 3.2.2. Effect of Reactant Concentrations

In order to optimize the reagent concentrations for the selective determination of Co(II), the Taguchi experimental design method was used. Different reagent concentrations were tested using sequence 8, both in the absence and presence of interfering Cu(II) ions.

**Figure 1:**
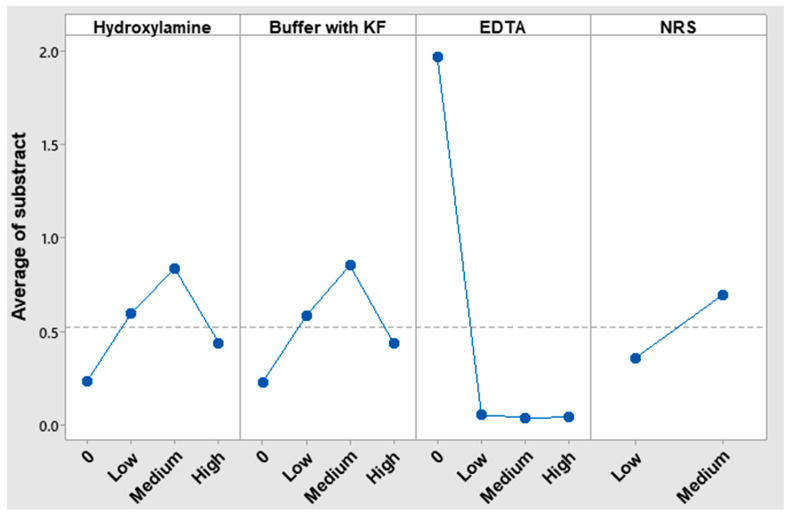
Taguchi analysis results for different concentration levels of each reagent.

The absorbance values obtained from the tests with and without Cu(II) were subtracted to assess the net interference effect, and the results were analyzed using the Taguchi signal-to-noise (S/N) ratio, applying the ‘nominal-is-best’ criterion with a target value of zero. This analysis was carried out using Minitab 19 software. Figure 1 presents the corresponding S/N analysis results graphically, while Table S4 summarizes the absorbance values obtained under the Taguchi experimental design conditions.

EDTA played a pivotal role since its presence significantly reduced interferences. The response value in the Taguchi analysis of the low concentration level was nearly zero, indicating that there was sufficient EDTA to eliminate interferences. However, the medium concentration level, which has been utilized thus far, was maintained to ensure that it was in excess.

In the case of NRS, the low-level concentration of it was closest to zero. One possible explanation is that reducing the NRS concentration enhances the method’s selectivity. However, it is also plausible that this is due to fewer complexes being formed, which would reduce the absorbances of the samples with and without copper, bringing their values closer together. To verify the cause, Section 3.4 presents two absorbance scans of each metal coordinated with NRS: (i) one using the low concentration and (ii) another using the medium concentration.

The absence of KF in acetate–acetic acid buffer was the best option to avoid interference caused by Cu(II). Nevertheless, the KF in acetate–acetic buffer was still employed due to the necessity of eliminating Fe(III) interference and keeping pH at optimal conditions for reaction.

Hydroxylamine was counterproductive in removing Cu(II) interference as in its absence the nominal value is near to 0. Therefore, the resulting Cu(I) from the reduction caused by hydroxylamine interferes as much as Cu(II).

Consequently, in the instance of Cu(II), the addition of EDTA in excess subsequent to NRS, which concentration was the minimum as possible, was sufficient to avoid its interference.

### 3.3. Study of Fe(III) Interferences

A new set of experiments was conducted to evaluate the effect of Fe(III) interference using a concentration of 16 mg L^−1^ (2.9 × 10^−4^ mol L^−1^) Fe(III) [60]. The study focused on testing the most promising reagent sequences identified in the Cu(II) interference experiments, as well as exploring additional sequences to deepen the understanding of the system’s behavior. The results are summarized in Table 6.

The results presented in Table 6 show the effect of 16 mg L^−1^ (2.9 × 10^−4^ mol L^−1^) Fe(III) on the absorbance signal of the NRS–Co(II) complex for various sequences of reagent addition. The absolute and relative errors (based on the difference in absorbance with and without Fe(III)) were used to evaluate the degree of interference. A lower relative error indicates better selectivity and effective masking of Fe(III).

Sequences 1, 2, 8, 9, 10, 11, and 12 resulted in large absolute and relative errors, with relative errors ranging from 154.1% to 302.6%, indicating that Fe(III) strongly interferes under these reagent addition orders. These sequences begin with NRS or hydroxylamine and delay the addition of EDTA and/or KF in acetate–acetic acid buffer.

In contrast, sequences 13, 15, and 16 demonstrated minimal interference, with relative errors between 12.3% and 19.6%, indicating effective masking of Fe(III). These sequences begin with KF in acetate–acetic acid buffer and add NRS early, before or immediately after hydroxylamine. The best result was obtained with sequence 13 (B–H–N–E), which yielded only a 12.3% relative error. This suggests that initiating the sequence with KF in acetate–acetic acid buffer effectively sequesters Fe(III). These observations align with the known high affinity of Fe(III) for fluoride and reinforce the importance of masking agent position in the addition sequence.

Additionally, the use of hydroxylamine was also counterproductive in this experiment. Thus, it was tested when hydroxylamine was not used in sequences 13, 15 and 16, which means that all three sequences converge into a single sequence. The results, which were shown in the Supplementary Information in section “Effect of Hydroxylamine”, proved that it is better not to use hydroxylamine. The measured absorbances are presented in Table S5.

### 3.4. Absorbance Spectrum of Metal–NRS Complexes

To further improve the selectivity of the proposed method, absorbance spectra of various metal–NRS coordination complexes were recorded. The main objective was to identify wavelengths at which Co(II) can be selectively determined, minimizing interference from other metal ions with overlapping absorbance.

To expand on the findings presented in Section 3.2.2—where a lower NRS concentration led to a lower Cu(II) signal—each metal complex was analyzed using two NRS concentrations in the stock solution: 2 g L^−1^ (0.0053 mol L^−1^) and 4 g L^−1^ (0.0106 mol L^−1^). Only 2 mL of the NRS solution was added and made up to 10 mL with Milli-Q water. Therefore, the final NRS concentration in mixture were 0.00106 mol L^−1^ and 0.0021 mol L^−1^, respectively.

The concentrations used were 10 mg L^−1^ (1.7 × 10^−4^ mol L^−1^) for Co(II) and 16 mg L^−1^ for (2.9 × 10^−4^ mol L^−1^) Fe(II), (2.9 × 10^−4^ mol L^−1^) Fe(III), (2.5 × 10^−4^ mol L^−1^) Cu(II) and (2.7 × 10^−4^ mol L^−1^) Ni(II).

**Figure 2:**
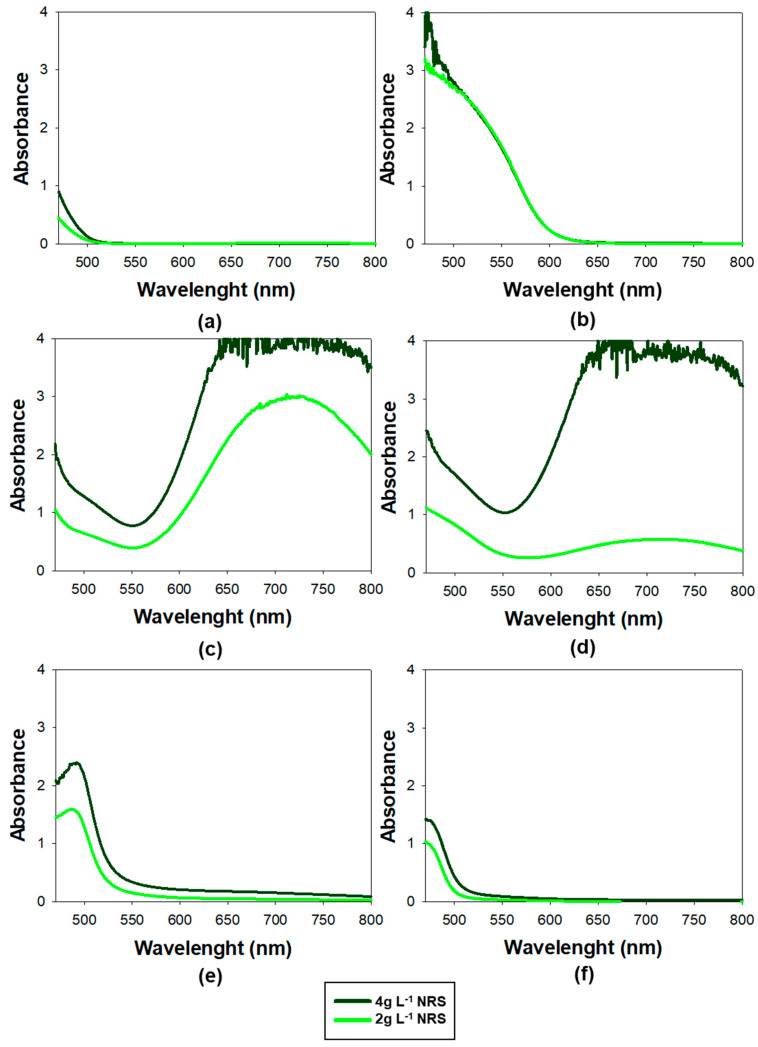
Absorbance spectra from different metal-NRS complexes using UV-Vis spectrophotometry, scanned from 470 to 800 nm. (**a**) NRS only, (**b**) Co(II), (**c**) Fe(II), (**d**) Fe(III), (**e**) Cu(II), (**f**) Ni(II).

Spectral scans were conducted across the range of 470 to 800 nm, using Milli-Q water as the blank. The resulting spectra are presented in Figure 2.

The optimum range for measuring the absorbance of the Co(II)-NRS complex was at 525 nm, as this was the wavelength with the highest absorption at which the NRS showed no absorbance. It was consistent with the findings of previous studies [22,29,41].

Moreover, it can be seen in Figure 2 that NRS–cobalt complex exhibits high absorbance in the 525–560 nm range. In fact, at 550 nm was particularly significant for enhancing the method’s selectivity, as the nickel and copper complexes practically did not absorb, meaning their interference in the determination of cobalt will be negligible. Additionally, the Fe(II) and Fe(III) complexes show a minimum in absorbance at 550 nm, further minimizing potential interference at this wavelength. These findings suggested that spectral interferences were significantly lower at 550 nm than at 525 nm, making 550 nm a more suitable wavelength for accurate cobalt determination. The results confirmed that using lower concentrations of NRS lowered the absorbance of the different metals except for Co(II), which remained unchanged.

An additional noteworthy observation was the absorbance spectrum of Fe(II), which displayed a high and wide peak around 710 nm. This was particularly important because, as previously highlighted, it posed the greatest challenge in terms of interference. To monitor its potential interference, 710 nm was employed as a sentinel wavelength: if absorbance was detected at this wavelength, it strongly suggested that there were NRS-Fe(II) or NRS-Fe(III) and may also be contributing to the signal at 550 nm. This approach served as an early warning system to identify possible Fe(II) interference and ensure the selectivity of the method for Co(II).

At this point, absorbance measured was 550 nm and 710 nm, which was the sentinel peak. The concentration of NRS was 2 g L^−1^.

### 3.5. Study of Fe(II) Interferences

Once the optimal reagent addition sequence, the best combination of reagent concentrations, and the most favorable wavelengths for the analysis were established, the interference of Fe(II)—which is the most challenging interference to address—was investigated. Moreover, it is important to consider that Fe(II) ions can be present at high concentrations in the bioleachate from batteries. To tackle this issue, an evaluation was conducted to determine whether increasing the volume of KF in acetate–acetic acid buffer could completely mask the Fe(II). Samples containing only Fe(II) were prepared, with 4, 5, 6, and 7 mL of KF in acetate–acetic acid buffer added to each. All experiments were conducted in triplicate. The molar concentrations of KF in acetate–acetic acid buffer are in Chemicals and Methods, Section Study of Fe(II) interferences.

As previously observed, reducing the concentration of NRS decreases the signal caused by Fe(II). Therefore, 1mL of NRS at 4g L^−1^ (0.0106 mol L^−1^) was used in these tests instead of the 2 mL employed in earlier experiments. As a result, the NRS concentration in mixture was 0.00106 mol L^−1^.

Additionally, adjustments were made to the volume and concentration of the EDTA solution. Instead of using 2 mL of a 0.05 mol L^−1^ solution, 1 mL of a 0.1 mol L^−1^ solution was employed. Therefore, the EDTA concentration in mixture was 0.01 mol L^−1^.

These changes provided greater flexibility to vary the volumes of other reagent solutions added while maintaining a total volume of 10 mL and allowing to perform the test adding 7 mL of KF in acetate–acetic acid buffer. Importantly, this modification did not result in significant changes in the outcomes.

Finally, to work with more representative laboratory conditions, a Fe(II) concentration of 20 mg L^−1^ (3.6 × 10^−4^ mol L^−1^) was used, as opposed to the 16 mg L^−1^ (2.9 × 10^−4^ mol L^−1^) utilized in previous experiments based on the reference [41]. This concentration better reflects the Fe(II) concentration in real samples analyzed.

The results were shown in Table 7. The obtained values indicated that the signal progressively decreased as more volume of KF in acetate–acetic acid buffer was added. However, beyond a volume of 6 mL, the signal no longer decreases and remains at a residual level. Therefore, it was concluded that this strategy is insufficient to avoid the interference caused by 20 mg L^−1^ of Fe(II).

Nevertheless, the issue was surmounted by the addition of 4 mL of 25% *v/v* H_2_SO_4_ at the end of the sequence, followed by a waiting period of 10 min, as detailed in the subsequent section.

### 3.6. Study to Remove Turbidity from Fe(OH)_3_ and Al(OH)_3_

During the evaluation of the conditions optimized thus far, it was observed that the addition of KF in acetate–acetic acid buffer produced turbidity in the presence of 20 mg L^−1^ Fe(III) (3.6 × 10^−4^ mol L^−1^) and 30 mg L^−1^ Al(III) (0.0011 mol L^−1^). This phenomenon was attributed to the potential formation of Fe(OH)_3_ and Al(OH)_3_. This issue had not been detected earlier, as aluminum had not been previously tested, and 16 mg L^−1^ (2.9 × 10^−4^ mol L^−1^) of Fe(III) did not generate observable turbidity. However, at 20 mg L^−1^ Fe(III), the effect became evident.

To address this, the volume added of KF in acetate–acetic acid buffer was reduced to 1 mL (0.057 mol L^−1^ of KF, 0.0022 mol L^−1^ of acetate, 7 × 10^−4^ mol L^−1^ of acetic acid in the mixture), but turbidity still occurred. In the case of Fe(III), the problem was resolved by adding 1 mL of H_2_SO_4_ solution adjusted to pH 1.8 before the addition of KF in acetate–acetic acid buffer. However, this introduced variability in absorbance between replicates, probably due to the new addition between the sample and the KF in acetate–acetic acid buffer. This issue was resolved by shaking the solution immediately after the addition of the KF-buffer mixture. Under these conditions, absorbance was consistently zero at two monitored wavelengths, 550 and 710 nm.

The final working protocol was as follows: sample, 1 mL of solution adjusted to pH 1.8 with H_2_SO_4_, 1 mL of KF in acetate–acetic acid buffer, vigorous shaking, 2 mL of NRS (at 2 g L^−1^), 1 mL of EDTA, 4 mL of Milli-Q water and diluted to a final volume of 10 mL with Milli-Q water.

Using this protocol, Al(III) was tested at 30 mg L^−1^, and it exhibited behavior similar to Fe(III): turbidity appeared upon the addition of KF in buffer. To mitigate this, the 4 mL of Milli-Q water added at the end were replaced with H_2_SO_4_ solutions at increasing concentrations (5%, 10%, 15%, 20%, and 25% *v*/*v*). A concentration of 5 % H_2_SO_4_ was sufficient to eliminate the turbidity caused by Al(III). As discussed in Section 3.1.2, concentrated acids dissociate all metal complexes except Co(II)–NRS. This allowed for the potential removal of Fe(II) interference. To confirm this, the absorbance of Fe(II) was monitored at 550 and 710 nm after the addition of H_2_SO_4_ at 10% and 25% *v*/*v*. At 25%, no signal was detected after 10 min; at 10%, the signal disappeared after 30 min (see Figure S4).

### 3.7. Optimal Method

After conducting all the necessary studies to minimize interferences caused by metal ions in the colorimetric determination of cobalt using the complexing reagent NRS, the optimized method has been established as follows:

1. Sample;
2. 1 mL solution adjusted to pH 1.8 with H_2_SO_4_;
3. 1 mL of solution containing 33.3 g L^−1^ (0.57 mol L^−1^) KF in 3 g L^−1^ sodium acetate trihydrate (0.022 mol L^−1^)—acetic acid (0.007 mol L^−1^) buffer;
4. Shake the solution;
5. 2 mL of 2 g L^−1^ (0.0053 mol L^−1^) NRS with 3 g L^−1^ sodium acetate trihydrate (0.022 mol L^−1^);
6. 1 mL of EDTA 0.1 mol L^−1^;
7. 4 mL H_2_SO_4_ at 25% *v*/*v;*
8. Made up to a final volume of 10 mL with Milli-Q water.

Hydroxylamine was not used in this final version, and the amount of concentrated acid necessary was reduced.

The method was tested using all major metal ions found in battery bioleachates. No interferences were observed at 550 and 710 nm even at concentrations higher than those typically found in real samples, as shown in Table S6.

### 3.8. Method Validation

To evaluate the reliability and selectivity of the method for Co(II) determination in battery bioleachate samples, a validation study was carried out. The parameters assessed included precision (expressed as repeatability), limit of detection (LoD), limit of quantification (LoQ), linearity, accuracy, and application to real samples. The validation was conducted using measurement at a wavelength of 550 nm.

#### 3.8.1. Repeatability

To assess the method’s precision, six replicate analyses were performed on solutions containing 2.5 and 10 mg L^−1^ of Co(II). The close consistency of the results demonstrates good repeatability. The data are summarized in Table 8.

The coefficient of variation (CV or RSD%) ranged from 1.70% to 2.65%.

These repeatability values are considered acceptable according to the Horwitz criterion [56].

#### 3.8.2. Analysis of Real Samples

In order to ensure the robustness and reliability of the proposed method, a validation was conducted using real samples with complex matrices. The mediums examined were the *Acidithiobacillus ferrooxidans* culture medium, water from the Agulla reservoir in Manresa, bioleachate from smartphone PCBs, and bioleachate from bike and scooter batteries. It was noteworthy that, aside from the battery bioleachate containing Co(II), Co(II) had to be added to the other mediums since it was not inherently present. All measures were performed in triplicate.

All samples, except for the reservoir water, were diluted 450 times. UV-Vis analyses results were compared with those obtained using atomic absorption spectroscopy. The concentration values obtained from both methods were found to be nearly identical, as presented in Table 9. This close agreement between the results reinforces the reliability and robustness of the developed method, highlighting its suitability for selective Co(II) determination in diverse and complex sample matrices and presence of interferers.

#### 3.8.3. Accuracy

Accuracy, defined as the closeness of the experimental results to the true value, was assessed in terms of recovery (RV%) of a known amount of added standard. To this end, a known concentration of Co(II) (5.5 mg L^−1^) was spiked into bioleachate samples obtained from bike and scooter batteries. As it can be seen in Table 10, the recovery values were close to 100%, indicating that the method provides accurate results. All the measurements were performed in triplicate.

#### 3.8.4. Lineal Range

**Figure 3:**
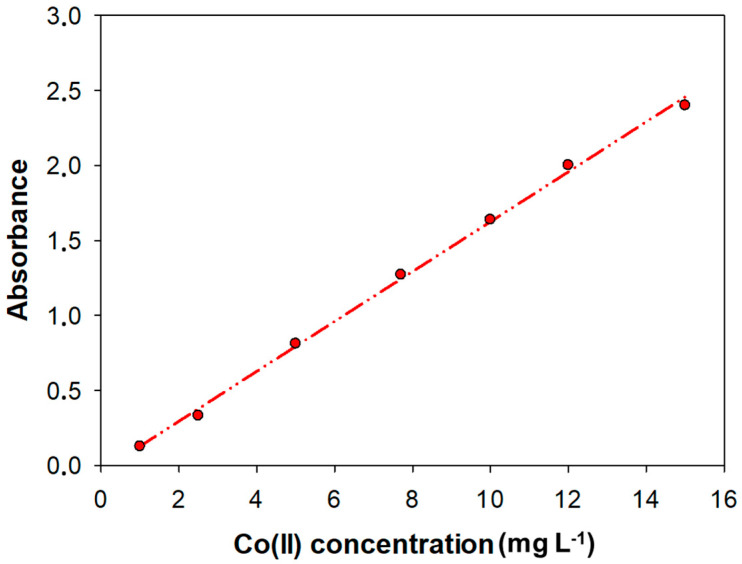
Calibration curves and linear adjustment within the linear range for the determination of Co(II). The equation was Abs = (0.166 ± 0.003) [Co(II)]—(0.04 ± 0.03), R^2^ = 0.998.

The linearity of an analytical method represents the relationship between analyte concentration and the analytical signal obtained. The linear response range, defined as the interval between the upper limit of quantification (LoQ) to the highest concentration, was determined by analyzing standard solutions ranging from 1 to 20 mg L^−1^. Thus, the calibration curve obtained was calculated by least squares. The upper limit was 15 mg L^−1^ and R^2^ was 0.998. The calibration plot is shown in Figure 3. The slope was 0.166 L mg^−1^.

#### 3.8.5. Limit of Detection and Limit of Quantification

The limit of detection (LoD) and limit of quantification (LoQ) were determined by signal-to-noise approach. The signal-to-noise ratio was established by measuring the signals of 5 blanks, which contained all reagents without any metal. The LoD and LoQ were then calculated using the mean (X) and standard deviation (S_b_) values, according to [57]: LoD = (X + 3S_b_)/S and LoQ = (X + 10S_b_)/S, where S_b_ is the standard deviation of the blank mean and S was the slope of the calibration curve or sensitivity. Using this method, the LoD was 0.1 mg L^−1^ and the LoQ was 0.3 mg L^−1^.

## 4. Conclusions

A simple, selective, and cost-effective spectrophotometric method, with no previous extraction and boiling step, was developed and validated for the determination of Co(II) in battery bioleachates. The optimization of the reagent addition sequence (sample, H_2_SO_4_ at pH = 1.8, KF in acetate–acetic acid buffer, shake the solution, NRS, EDTA and H_2_SO_4_ at 25% *v*/*v*), reagent concentrations, and measurement wavelength (550 nm) significantly improved selectivity of the method.

Particular attention was given to minimizing interferences, especially from Fe(II), which was effectively masked through the strategic use of potassium fluoride and EDTA under acidic conditions.

Absorbance spectra of various metal-NRS complexes among 480 and 800 nm confirmed a high absorbance from the complexation of Co(II) with the NRS at 525 and 550 nm. In contrast, absorption of the other metal complexes at 550 nm was negligible, in the case of Cu(II) and Ni(II), or minimal, in the case of Fe(II) and Fe(III). Thus, 550 nm was selected for selective Co(II) measurements even in the presence of interfering metal ions such as Fe(II) Fe(III), Cu(II) and Ni(II).

The method demonstrated excellent repeatability (RSD% < 2.7), low limits of detection and quantification, a wide linear response range, and high accuracy, with recovery values near 100% in real bioleachate samples.

Given its robustness, simplicity, and low cost, this method represents a valuable tool for routine monitoring of cobalt in hydrometallurgical processes, bioleachate from batteries and environmental samples derived from spent lithium-ion batteries.

## Figures and Tables

**Table 1:** Combinations of reactants addition sequences tested in the presence of Cu(II) in the determination of Co(II). Where H is hydroxylamine, N is NRS, E is EDTA and B is KF in acetate–acetic acid buffer.

	Position of Reagent			
Sequence Number	1st	2nd	3rd	4th
1	H	N	B	E
2	H	N	E	B
3	H	B	N	E
7	N	H	B	E
8	N	H	E	B
9	N	B	H	E
10	N	B	E	H
11	N	E	H	B
12	N	E	B	H
13	B	H	N	E
15	B	N	H	E
16	B	N	E	H

**Table 2:** Combinations of reactant addition sequences tested in the presence of Fe(III) in the determination of Co(II). Where H is hydroxylamine, N is NRS, E is EDTA and B is KF in acetate–acetic acid buffer.

	Position of Reagent			
Sequence Number	1st	2nd	3rd	4th
1	H	N	B	E
2	H	N	E	B
8	N	H	E	B
9	N	B	H	E
10	N	B	E	H
11	N	E	H	B
12	N	E	B	H
13	B	H	N	E
15	B	N	H	E
16	B	N	E	H

**Table 3:** All tested concentrations of metal interferents in bioleachate samples using the optimized experimental conditions. The concentrations are expressed in mg L^−1^ and mol L^−1^.

Metal	Low Concentration (mg L^−1^/mol L^−1^)	Medium Concentration (mg L^−1^/mol L^−1^)	High Concentration (mg L^−1^/mol L^−1^)
Mn(II)	5/9.1 × 10^−5^	10/1.8 × 10^−4^	20/3.6 × 10^−5^
Al(III)	20/7.4 × 10^−4^	30/0.0011	40/0.0015
Cu(II)	40/6.3 × 10^−4^	50/7.9 × 10^−4^	60/9.4 × 10^−4^
Ni(II)	60/0.0010	70/0.0012	80/0.0014
Fe(III)	10/1.8 × 10^−4^	20/3.6 × 10^−4^	30/5.4 × 10^−4^
Fe(II)	10/1.8 × 10^−4^	20/3.6 × 10^−4^	30/5.4 × 10^−4^

**Table 4:** Summary of the tested reagents, including their role, masking effect, order of addition, and associated limitations.

Reagent	Activity	Masking Efficiency	Effect of Order of Addition	Limitations
Nitroso-R-Salt (NRS)	Forms colored complexes with Co(II), Ni(II), Cu(II), Fe(II), Fe(III); red-orange with Co(II).	Not applied.	Needs to be added before the EDTA and acid.	Poor reactivity at acidic pH; excess NRS does not prevent Fe(II) interference; degrades over time unless protected from light or stabilized with acetate.
Strong Acids (HCl/H_2_SO_4_)	Destabilizes metal-NRS complexes except Co(II), improving selectivity at very low pH.	Indirect masking by degrading interfering complexes (Cu, Ni, Fe).	If it was added before the NRS the reaction requires almost 1 h to complete. After, the waiting time to dissociate Fe(II)-NRS was almost 30 min.	Requires large acid volume and long waiting times; safety and practicality issues.
Hydroxylamine	Intended as a reducing agent for Fe(III) and Cu(II).	None observed; reduction was ineffective and worsened interference.	Irrelevant due to poor performance.	No positive effect; exacerbates Fe interference.
Acetate	Regulates pH; slightly stabilizes NRS; accelerates Co(II) complex formation.	Weak and temporary masking of Fe(III) only when added before NRS; no effect on Cu(II) or Fe(II).	Addition before or after NRS accelerates the reaction; masking is only effective if added before NRS.	Ineffective masking agent overall; key role is pH control and NRS stabilization.
Fluoride (KF)	Enhance selectivity by masking Fe(II) and Fe(III).	Strong masking of Fe(II) and Fe(III) if added before NRS; no effect on Cu(II) and Ni(II).	Must be added before NRS to mask Fe; cannot reverse already-formed metal-NRS complexes.	Does not mask Cu(II) or Ni(II); ineffective if added after NRS.
EDTA	Chelates all metal ions except Co(II); dissociates previously formed complexes with most metals.	Effective masking of Cu(II), Ni(II) and Fe(III), if added after NRS; prevents NRS complexation if added first.	Must be added after NRS to preserve Co(II) complex and remove interferences.	Non-specific; terminates all NRS-metal reactions; addition sequence critical; premature addition halts all coordination.
Acetate (buffer) plus Fluoride	pH regulation plus Fe masking; simplified reagent delivery.	Maintains fluoride masking capacity; acetate contributes to Fe(III) masking and pH stability.	Order of buffer and fluoride does not matter as long as both precede NRS; combined into one solution.	Simplifies procedure but relies on fluoride for masking; acetate alone insufficient.

**Table 5:** Absorbance determined for the combinations tested in the addition of the order of the reagents in the case of 40 mg L^−1^ Cu(II) as interference and 1.6 mg L^−1^ Co(II). Where H is hydroxylamine, N is NRS, E is EDTA and B is KF in acetate–acetic acid buffer. Experimental uncertainty is the standard deviation (*n* = 3).

	Position of Reagent				Absorbance (λ = 525 nm)		Error	
Sequence Number	1st	2nd	3rd	4th	Without Cu(II)	With Cu(II)	Absolute Error	Relative Error (%)
1	H	N	B	E	0.383 ± 0.008	0.301 ± 0.019	0.082	21.4
2	H	N	E	B	0.30 ± 0.03	0.280 ± 0.008	0.016	5.3
3	H	B	N	E	0.371 ± 0.016	0.301 ± 0.017	0.071	19.1
7	N	H	B	E	0.35 ± 0.07	0.413 ± 0.019	0.062	17.7
8	N	H	E	B	0.4144 ± 0.0025	0.420 ± 0.012	0.006	1.4
9	N	B	H	E	0.42 ± 0.03	0.45 ± 0.10	0.028	6.7
10	N	B	E	H	0.4220 ± 0.0021	0.428 ± 0.003	0.006	1.4
11	N	E	H	B	0.398 ± 0.019	0.41 ± 0.03	0.013	3.3
12	N	E	B	H	0.41 ± 0.03	0.49 ± 0.06	0.078	19.0
13	B	H	N	E	0.371 ± 0.007	0.29 ± 0.04	0.078	21.0
15	B	N	H	E	0.40 ± 0.03	0.38 ± 0.08	0.022	5.5
16	B	N	E	H	0.37 ± 0.06	0.420 ± 0.017	0.048	13.0

**Table 6:** Absorbance determined for the combinations tested in the addition of the reagents in the case of 16 mg L^−1^ Fe(III). Where H is hydroxylamine, N is NRS, E is EDTA and B is KF in acetate–acetic acid buffer. Experimental uncertainty is the standard deviation (*n* = 3).

	Position of Reagent				Absorbance (λ = 525 nm)		Error	
Sequence Number	1st	2nd	3rd	4th	Without Fe(III)	With Fe(III)	Absolute Error	Relative Error (%)
1	H	N	B	E	0.3751 ± 0.0018	1.082 ± 0.004	0.707	188.5
2	H	N	E	B	0.209 ± 0.008	0.8414 ± 0.007	0.6324	302.6
8	N	H	E	B	0.408 ± 0.020	1.28 ± 0.06	0.868	212.7
9	N	B	H	E	0.472 ± 0.019	1.23 ± 0.09	0.758	160.6
10	N	B	E	H	0.427 ± 0.006	1.20 ± 0.07	0.77	180.3
11	N	E	H	B	0.416 ± 0.022	1.14 ± 0.10	0.729	175.2
12	N	E	B	H	0.425 ± 0.020	1.081 ± 0.004	0.655	154.1
13	B	H	N	E	0.3831 ± 0.0020	0.430 ± 0.022	0.047	12.3
15	B	N	H	E	0.413 ± 0.008	0.494 ± 0.011	0.081	19.6
16	B	N	E	H	0.44 ± 0.03	0.521 ± 0.006	0.08	18.2

**Table 7:** Absorbance of Fe(II)-NRS adding different volumes of KF in acetate–acetic acid buffer; [Fe(II)] is 20 mg L^−1^.

	550 nm			710 nm		
Volume	Average	SD	CV (%)	Average	SD	CV (%)
4 mL	0.282	0.012	4.31	2.004	0.048	2.37
5 mL	0.110	0.005	4.19	0.36	0.03	7.10
6 mL	0.076	0.021	27.59	0.131	0.052	39.64
7 mL	0.096	0.024	25.14	0.30	0.19	65.00

**Table 8:** Repeatability results for the method.

Concentration (mg L^−1^)	Average	SD	CV (%)
2.5	0.328	0.009	2.65
10	1.64	0.03	1.70

**Table 9:** Comparison of Co(II) analysis in real samples between proposed method in UV-vis spectrophotometry and reference method atomic absorption spectrometry. Experimental uncertainty is standard deviation (*n* = 3).

Sample	Co(II) Added (mg L^−1^)	Co(II) Found (mg L^−1^)	
		UV-Vis 550 nm	Atomic Absorption Spectroscopy
Bioleached PCBs	2	2.041 ± 0.019	2.07 ± 0.08
	3.5	3.50 ± 0.03	3.70 ± 0.18
	6	6.11 ± 0.04	6.095 ± 0.025
Acidithiobacillus ferrooxidans medium	3	2.96 ± 0.03	3.10 ± 0.13
	4	3.981 ± 0.013	4.09 ± 0.04
	7	6.66 ± 0.06	6.66 ± 0.07
Water from the Parc de l’Agulla reservoir in Manresa	1.5	1.62 ± 0.07	1.66 ± 0.03
	8	7.476 ± 0.019	7.51 ± 0.07
	9	8.54 ± 0.03	8.39 ± 0.12
Bioleachate from bicycle and scooter batteries	-	0.818 ± 0.010	0.777 ± 0.018

**Table 10:** Accuracy results for the method. Experimental error is expressed as standard deviation.

Dilution Ration	Co(II) in Sample (mg L^−1^)	Co(II) in Spiked Sample (mg L^−1^)	RV (%)
1:450	0.71 ± 0.03	6.19 ± 0.14	99.55
1:360	0.86 ± 0.03	6.28 ± 0.08	98.69
1:270	1.262 ± 0.009	6.76 ± 0.06	99.87

## Data Availability

The data presented in this study are available upon request from the corresponding authors. The data are not publicly available because the repository that is used to keep the data is a private one provided by the university.

## Supplementary Materials

The following supporting information can be downloaded at: https://www.mdpi.com/article/10.3390/nano15161264/s1, Table S1: All possible combinations of reactant addition sequences. Table S2: Concentration levels used for experiments in a Taguchi model. Table S3: Experiments performed within the framework of a Taguchi model. Figure S1: Color of different complexes metal-NRS. Figure S2: Evolution of absorbance at 525 nm over time during the formation of the Co(II)-NRS complex (60 mg L^−1^ Co(II), pH = 2). Figure S3: UV-Vis absorbance spectra of Co(II)–NRS and Fe(II)–NRS complexes at 15 mg L^−1^ and 7.5 mg L^−1^, respectively, recorded from 470 to 800 nm. The effect of EDTA and fluoride on the formation of the Fe(II)–NRS complex is compared. Table S4: Results of absorbance from experiments performed within the framework of a Taguchi model in presence of 40 mg L^−1^ Cu(II) as interference and 1.6 mg L^−1^ Co(II) with sequence 8 (NRS, hydroxylamine, EDTA, KF in acetic-acetate buffer). Table S5: Absorbance of various interfering cations without hydroxylamine. The concentrations used were Co(II) 1.6 mg L^−1^, Cu(II) 40 mg L^−1^ and Fe(III) 16 mg L^−1^. Figure S4: Time for the disappearance of Fe(II)-NRS absorbance one minute after the addition of acid. Table S6: Absorbance of interferents using the validated method.
