# Supplementary material for: Enhancing the Selectivity of Nitroso-R-Salt for the Determination of Co(II) in Lithium Bioleaching Recovery of Smartphone Batteries Using a Combinatorial Methodology Approach
Source: Nanomaterials (Basel). 2025 Aug 16;15(16):1264. doi: 10.3390/nano15161264 (PMC12389291; doi:10.3390/nano15161264)
Supplement: Supplementary file 1 [file nanomaterials-15-01264-s001.zip › nanomaterials-3718684-supplementary.pdf]

nanomaterials

Supplementary Information

*for*

# Enhancing the Selectivity of Nitroso-R-Salt for the Determination of Co(II) in Lithium Bioleaching Recovery of Smartphone Batteries Using a Combinatorial Methodology Approach

**David Ricart [1], Antonio David Dorado [1], Mireia Baeza [2,*] and Conxita Lao-Luque [1,*]**

[1] Department of Mining, Industrial and ICT Engineering, Escola Politècnica Superior d'Enginyeria de Manresa,Universitat Politècnica de Catalunya, Avinguda de les Bases de Manresa 61-73, 08240 Manresa, Spain; david.ricart.fort@upc.edu (D.R.); toni.dorado@upc.edu (A.D.D.)
[2] GENOCOV Research Group, Department of Chemistry, Faculty of Science, Edifici C-Nord, Universitat Autònoma de Barcelona, Carrer dels Til·lers, 08193 Bellaterra, Spain
* Correspondence: mariadelmar.baeza@uab.cat (M.B.); conxita.lao@upc.edu (C.L.-L.)

**Table S1.** All possible combinations of reactant addition sequences. Where H is hydroxylamine, N is NRS, E is EDTA and B is KF in acetic-acetate buffer.

| | Position of reagent | | | |
|---|---|---|---|---|
| Sequence number | 1st | 2nd | 3rd | 4th |
| 1 | H | N | B | E |
| 2 | H | N | E | B |
| 3 | H | B | N | E |
| 4 | H | B | E | N |
| 5 | H | E | N | B |
| 6 | H | E | B | N |
| 7 | N | H | B | E |
| 8 | N | H | E | B |
| 9 | N | B | H | E |
| 10 | N | B | E | H |
| 11 | N | E | H | B |
| 12 | N | E | B | H |
| 13 | B | H | N | E |
| 14 | B | H | E | N |
| 15 | B | N | H | E |
| 16 | B | N | E | H |
| 17 | B | E | H | N |
| 18 | B | E | N | H |
| 19 | E | H | N | B |
| 20 | E | H | B | N |
| 21 | E | N | H | B |
| 22 | E | N | B | H |
| 23 | E | B | H | N |

| | | | | |
|---|---|---|---|---|
| 24 | E | B | N | H |

**Table S2.** Concentration levels of stock solutions used for experiments in a Taguchi model.

| Reagent | Not added | Low concentration | Medium concentration | High concentration |
|---|---|---|---|---|
| Hydroxylamine | 0 | 5% w/V (0.7 mol L$^{-1}$) | 10% w/V (1.4 mol L$^{-1}$) | 20% w/V (2.8 mol L$^{-1}$) |
| NRS | - | 2 g L$^{-1}$ NRS (0.0053 mol L$^{-1}$)<br>1.5 g L$^{-1}$ $CH_3COONa{\cdot}3H_2O$<br>(0.011 mol L$^{-1}$ of acetate) | 4 g L$^{-1}$ NRS (0.0106 mol L$^{-1}$)<br>3 g L$^{-1}$ $CH_3COONa{\cdot}3H_2O$<br>(0.022 mol L$^{-1}$ of acetate) | - |
| Buffer with KF | 0 | 1.5 g L$^{-1}$ $CH_3COONa{\cdot}3H_2O$<br>(0.011 mol L$^{-1}$ of acetate)<br>16.65 g L$^{-1}$ KF (0.29 mol L$^{-1}$)<br>0.0035mol/L acetic acid | 3 g L$^{-1}$ $CH_3COONa{\cdot}3H_2O$<br>(0.022 mol L$^{-1}$ of acetate)<br>33.3 g L$^{-1}$ KF (0.57 mol L$^{-1}$)<br>0.007mol/L acetic acid | 3 g L$^{-1}$ $CH_3COONa{\cdot}3H_2O$<br>(0.044 mol L$^{-1}$ of acetate)<br>66.6 g L$^{-1}$ KF (1.14 mol L$^{-1}$)<br>0.014mol/L acetic acid |
| EDTA | 0 | 0.05 mol L$^{-1}$ | 0.1 mol L$^{-1}$ | 0.2 mol L$^{-1}$ |

**Table S3.** Experiments performed within the framework of a Taguchi model.

| Experiment number | NRS | Hydroxylamine | EDTA | Buffer with KF |
|---|---|---|---|---|
| 1 | Low | 0 | 0 | 0 |
| 2 | Low | 0 | Low | Low |
| 3 | Medium | 0 | Medium | Medium |
| 4 | Medium | 0 | High | High |
| 5 | Medium | Low | Low | 0 |
| 6 | Medium | Low | 0 | Low |
| 7 | Low | Low | High | Medium |
| 8 | Low | Low | Medium | High |
| 9 | Low | Medium | Medium | 0 |
| 10 | Low | Medium | High | Low |
| 11 | Medium | Medium | 0 | Medium |
| 12 | Medium | Medium | Low | High |
| 13 | Medium | High | High | 0 |
| 14 | Medium | High | Medium | Low |
| 15 | Low | High | Low | Medium |
| 16 | Low | High | 0 | High |

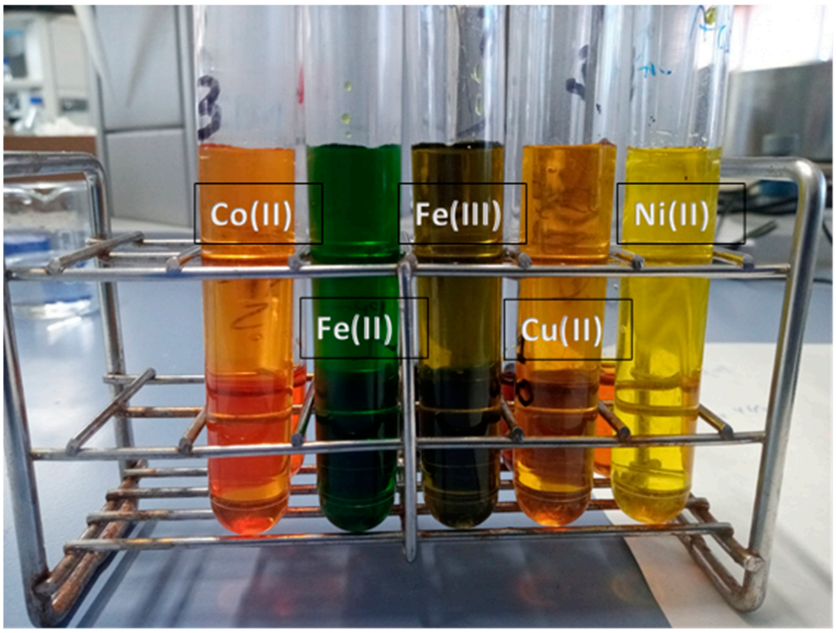

**Figure S1.** Color of different complexes metal-NRS.

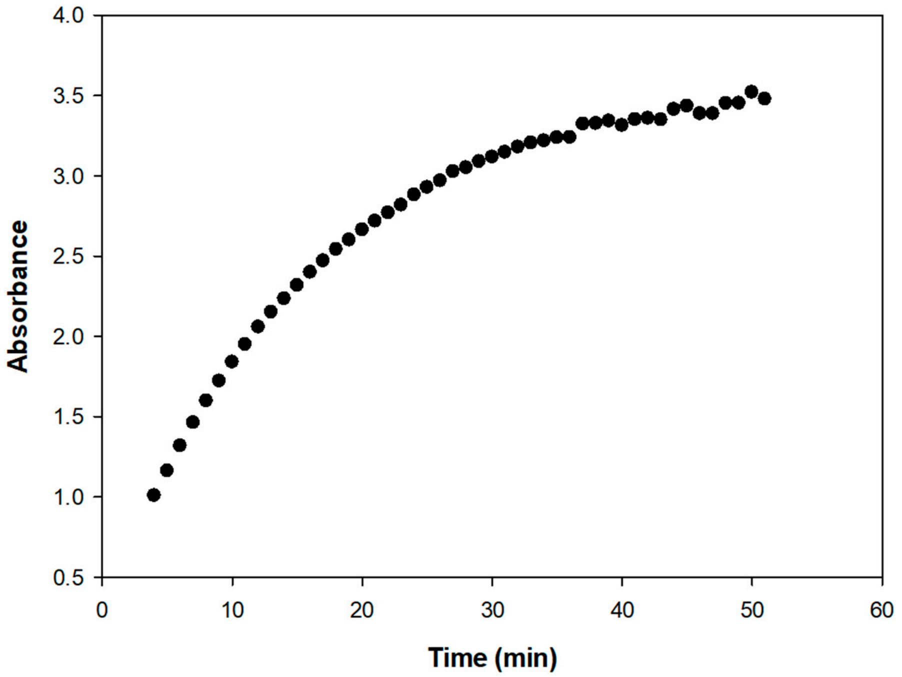


**Figure S2.** Evolution of absorbance at 525 nm over time during the formation of the Co(II)-NRS complex (60 mg $L^{-1}$ Co(II), pH = 2).

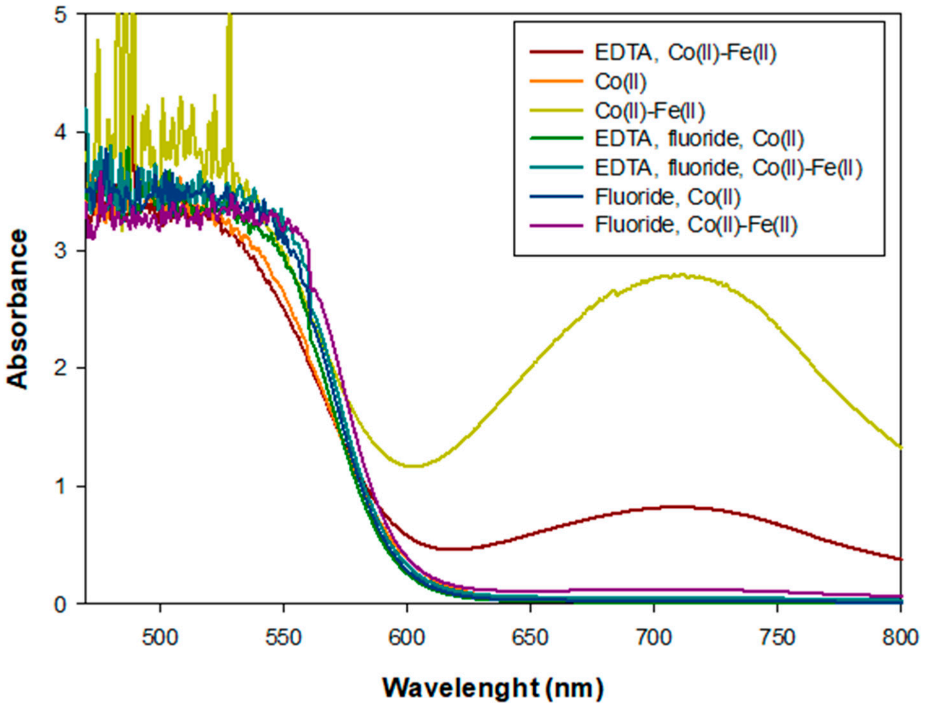


**Figure S3.** UV-Vis absorbance spectra of Co(II)–NRS and Fe(II)–NRS complexes at 15 mg $L^{-1}$ and 7.5 mg $L^{-1}$, respectively, recorded from 470 to 800 nm. The effect of EDTA and fluoride on the formation. of the Fe(II)–NRS complex is compared. The spectra illustrate that while Co(II) consistently forms its complex with NRS, the presence of EDTA and fluoride significantly influences the coordination of Fe(II) with NRS. Fluoride was added before NRS and EDTA after it.

**Table S4.** Results of absorbance from experiments performed within the framework of a Taguchi model in presence of 40 mg L$^{-1}$ Cu(II) as interference and 1.6 mg L$^{-1}$ Co(II) with sequence 8 (NRS, hydroxylamine, EDTA, KF in acetic-acetate buffer). Experimental uncertainty is the standard deviation ($n$=3).

| | Concentrations of reagent | | | | Results | | |
|---|---|---|---|---|---|---|---|
| **Experiment number** | **NRS** | **Hydroxylamine** | **EDTA** | **KF in buffer** | **Without Cu(II)** | **With Cu(II)** | **Subtract** |
| 1 | Low | 0 | 0 | 0 | 0..373 ± 0.022 | 1.173 ± 0.025 | 0.8000 |
| 2 | Low | 0 | Low | Low | 0.419 ± 0.004 | 0.344 ± 0.010 | 0.0752 |
| 3 | Medium | 0 | Medium | Medium | 0.425 ± 0.010 | 0.45 ± 0.03 | 0.0223 |
| 4 | Medium | 0 | High | High | 0.410 ± 0.014 | 0.387 ± 0.005 | 0.0223 |
| 5 | Medium | Low | Low | 0 | 0.408 ± 0.004 | 0.393 ± 0.007 | 0.0154 |
| 6 | Medium | Low | 0 | Low | 0.19 ± 0.03 | 2.41 ± 0.24 | 2.2227 |
| 7 | Low | Low | High | Medium | 0.433 ± 0.010 | 0.34 ± 0.03 | 0.0925 |
| 8 | Low | Low | Medium | High | 0.435 ± 0.004 | 0.390 ± 0.011 | 0.0454 |
| 9 | Low | Medium | Medium | 0 | 0.194 ± 0.017 | 0.119 ± 0.008 | 0.0747 |
| 10 | Low | Medium | High | Low | 0.381 ± 0.005 | 0.355 ± 0.020 | 0.0267 |
| 11 | Medium | Medium | 0 | Medium | 0.435 ± 0.012 | 3.65 ± 0.20 | 3.2110 |
| 12 | Medium | Medium | Low | High | 0.371 ± 0.020 | 0.405 ± 0.010 | 0.0342 |
| 13 | Medium | High | High | 0 | 0.355 ± 0.010 | 0.34 ± 0.05 | 0.0161 |
| 14 | Medium | High | Medium | Low | 0.386 ± 0.011 | 0.393 ± 0.007 | 0.0078 |
| 15 | Low | High | Low | Medium | 0.38 ± 0.04 | 0.289 ± 0.008 | 0.0873 |
| 16 | Low | High | 0 | High | 0.49 ± 0.04 | 2.13 ± 0.07 | 1.6438 |

**Table S5.** Absorbance of various interfering cations without hydroxylamine. The concentrations used were Co(II) 1.6 mg L$^{-1}$, Cu(II) 40 mg L$^{-1}$ and Fe(III) 16 mg L$^{-1}$. Experimental uncertainty is the standard deviation ($n$=3).

| Sample | Absorbance |
|---|---|
| Co(II) | 0.41 ± 0.03 |
| Co(II) - Fe(III) | 0.443 ± 0.025 |
| Co(II) - Cu(II) | 0.436 ± 0.004 |

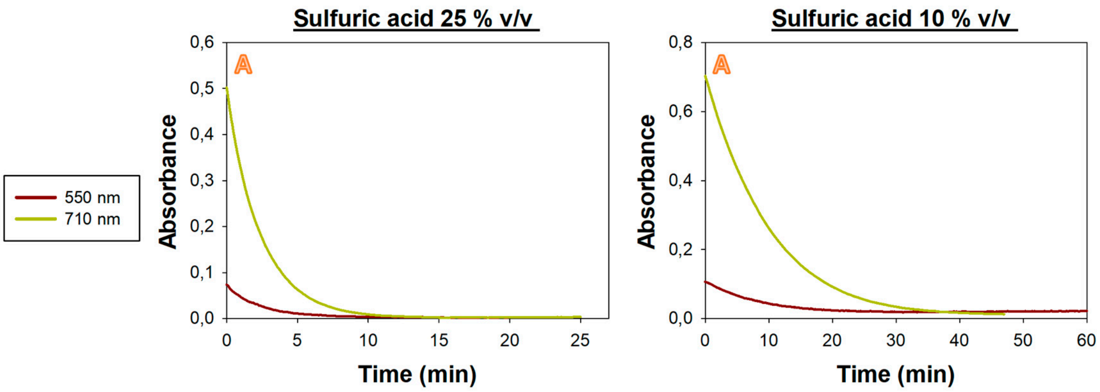


**Figure S4.** Time for the disappearance of Fe(II)-NRS absorbance one minute after the addition of acid.

**Table S6.** Absorbance of interferents using the validated method.

| Cation | Concentration (mg/L) | Abs 550 nm | Abs 710 nm |
| --- | --- | --- | --- |
| Mn(II) | 5 | -0.0140 | -0.0127 |
| | 10 | -0.0023 | -0.0023 |
| | 20 | -0.0250 | -0.0203 |
| Al(III) | 20 | -0.0217 | -0.0170 |
| | 30 | -0.0110 | -0.0077 |
| | 40 | -0.0187 | -0.0140 |
| Cu(II) | 40 | -0.0193 | -0.0113 |
| | 50 | -0.0107 | -0.0027 |
| | 60 | -0.0180 | -0.0067 |
| Ni(II) | 60 | -0.0227 | -0.0157 |
| | 70 | -0.0153 | -0.0083 |
| | 80 | -0.0310 | -0.0210 |
| Fe(III) | 10 | -0.0200 | -0.0153 |
| | 20 | -0.0167 | -0.0120 |
| | 30 | -0.0280 | -0.0207 |
| Fe(II) | 10 | -0.0257 | -0.0200 |
| | 20 | -0.0143 | -0.0123 |
| | 30 | -0.0240 | -0.0220 |

## Effect of Hydroxylamine

Since all previous experiments indicated that hydroxylamine is counterproductive, this was so evident in sequence 16 in section 3.3, where adding hydroxylamine at the end resulted in a slight green colour, caused by the formation of NRS-Fe(II) complex, sequences 13, 15, and 16 were tested without it. However, omitting hydroxylamine caused all three sequences to converge into a single sequence: 1 mL of sample, 4 mL of buffer with KF, 2 mL of NRS, 2 mL of EDTA, and finally, 1 mL of deionized water to reach a total volume of 10 mL. The results are presented in Table S5. The used concentration was Co(II) 1.6 mg $L^{-1}$, Cu(II) 40 mg $L^{-1}$ and Fe(III) 16 mg $L^{-1}$.

It is clearly seen how the selective determination of Co(II) improves with respect to the previous experiments. Therefore, from this point on, hydroxylamine is no longer used.
